# Inclusion Body in a Leukocyte in Crimean-Congo Hemorrhagic Fever

**DOI:** 10.4274/tjh.2014.0284

**Published:** 2015-08-01

**Authors:** Zahit Bolaman, İrfan Yavaşoğlu, Gürhan Kadıköylü

**Affiliations:** 1 Adnan Menderes University Faculty of Medicine, Division of Hematology, Aydın, Turkey

**Keywords:** Crimean-Congo hemorrhagic fever, leukocyte

A 49-year-old male patient was admitted to the emergency room with fever for 5 days and nasal bleeding. A tick bite was learned to have occurred 10 days before. On physical examination, the patient was confused, with a fever of 39 °C and widespread petechiae. In his laboratory examination, hemoglobin was 9 g/dL, WBC count was 16,000/mm3, and platelet count was 8000/mm3. In Giemsa-stained peripheral blood smear, rare schistocytes and platelets, 80% neutrophils, 18% lymphocytes, and 2% monocytes were observed. In the cytoplasm of a lymphomonocytic cell, a blue-violet inclusion body was seen (Figure 1). Informed consent was obtained. The patient was prediagnosed with Crimean-Congo hemorrhagic fever (CCHF) and accordingly administered fresh frozen plasma and platelet therapy. The patient was lost after massive gastrointestinal bleeding as a result of multiple organ failure. A positive PCR result was obtained for CCHF. Inclusion bodies in leukocytes can be seen in congenital diseases, hematological malignancies, infections (especially viral), burns, and pregnancy and after cytotoxic therapy. To the best of our knowledge, in the diagnosis of CCHF, inclusion bodies in leukocytes have not been reported previously in the literature. We suggest that they be remembered as a potential clue in diagnosis during peripheral blood smear examination.

**Conflict of Interest Statement**

The authors of this paper have no conflicts of interest, including specific financial interests, relationships, and/or affiliations relevant to the subject matter or materials included.

## Figures and Tables

**Figure 1:**
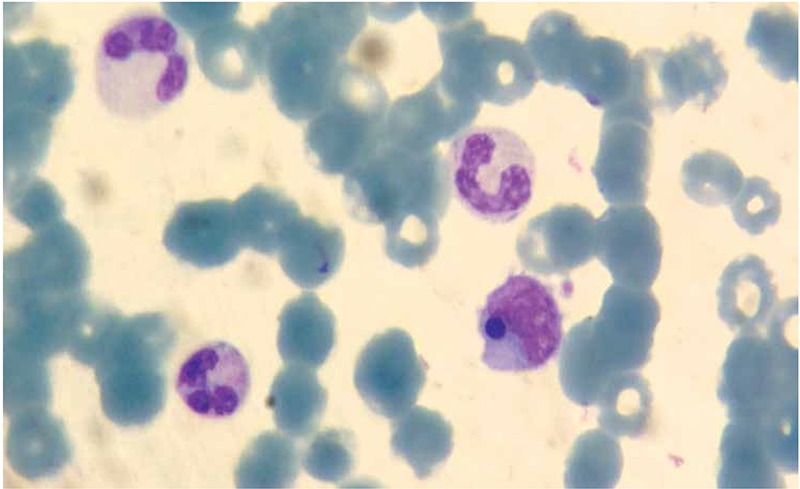
In the cytoplasm of a lymphomonocytic cell, a blue-violet inclusion body was seen.

